# Modeling the Microstructure Curvature of Boron-Doped Silicon in Bulk Micromachined Accelerometer

**DOI:** 10.3390/ma6010244

**Published:** 2013-01-15

**Authors:** Wu Zhou, Huijun Yu, Bei Peng, Huaqin Shen, Xiaoping He, Wei Su

**Affiliations:** 1 School of Mechatronics Engineering, University of Electronic Technology and Science of China, Chengdu 611731, China; E-Mails: yuhjuestc@126.com (H.Y.); beipeng@uestc.edu.cn (B.P); shq0520@yahoo.cn (H.S.); 2 Institute of Electronic Engineering, China Academy of Engineering Physics, Mianyang 621900, China; E-Mails: hxpiee@263.net (X.H.); zhouwu916@163.com (W.S.)

**Keywords:** microstructure, boron-doped silicon, multilayer solid model, curvature

## Abstract

Microstructure curvature, or buckling, is observed in the micromachining of silicon sensors because of the doping of impurities for realizing certain electrical and mechanical processes. This behavior can be a key source of error in inertial sensors. Therefore, identifying the factors that influence the buckling value is important in designing MEMS devices. In this study, the curvature in the proof mass of an accelerometer is modeled as a multilayered solid model. Modeling is performed according to the characteristics of the solid diffusion mechanism in the bulk-dissolved wafer process (BDWP) based on the self-stopped etch technique. Moreover, the proposed multilayered solid model is established as an equivalent composite structure formed by a group of thin layers that are glued together. Each layer has a different Young’s modulus value and each undergoes different volume shrinkage strain owing to boron doping in silicon. Observations of five groups of proof mass blocks of accelerometers suggest that the theoretical model is effective in determining the buckling value of a fabricated structure.

## 1. Introduction

Microstructure curvature was first observed in the surface machining of silicon that was doped with boron for realizing certain electrical and mechanical processes. Therefore, previous studies on buckling generally focused on structures with thin boron-doped silicon layers, such as diaphragms [1,2], thin films [3,4] and membranes [5]. However, with the increasing demand for microdevices with high sensitivity and high performance, bulk micromachining has been developed to create microstructures with thick layers similar to those achieved by the bulk silicon-dissolved wafer process, based on the self-stopped etch technique of heavy boron-doped silicon [6]. This fabrication technology is widely used to make microaccelerometers [7,8], microgyroscopes [9,10], microswitches [11,12], and microgears [13]. This technology also exhibits curvature behavior because its boron-doping mechanism is the same as that used in surface micromachining [5]. However, a difference exists. In the former, boron diffusion requires a longer time to realize a thick structure after self-stopped etching in EDP (Ethylene Diamine Pyrocatechol) [3]; accordingly, it is considered that the main contribution to buckling is the internal stress induced by volume shrinking caused by boron doping [5] rather than the thermal stress, which tends to show a uniform profile after annealing in the drive-in process. In this study, an analytical method based on a multilayer model is proposed to quantitatively determine the buckling of a bulk silicon structure that has a boron profile following a Gaussian function through the thickness [14]. This method is validated through experiments using five groups of sensors.

The bulk-dissolved wafer process (BDWP) is first introduced to show the formation of the boron-doped silicon structure. The material distribution characteristics are determined and considered as the main sources of curvature. Accordingly, a multilayer model is proposed to model this composite material structure in Section 2. Section 3 investigates groups of accelerometers fabricated through BDWP to verify the multilayer model with simple supports. The obtained results indicate that this approximate method is suitable for calculating the curvature of a BDWP structure. The conclusions and discussions are presented in Section 4.

## 2. Process and Model

**Figure 1:**
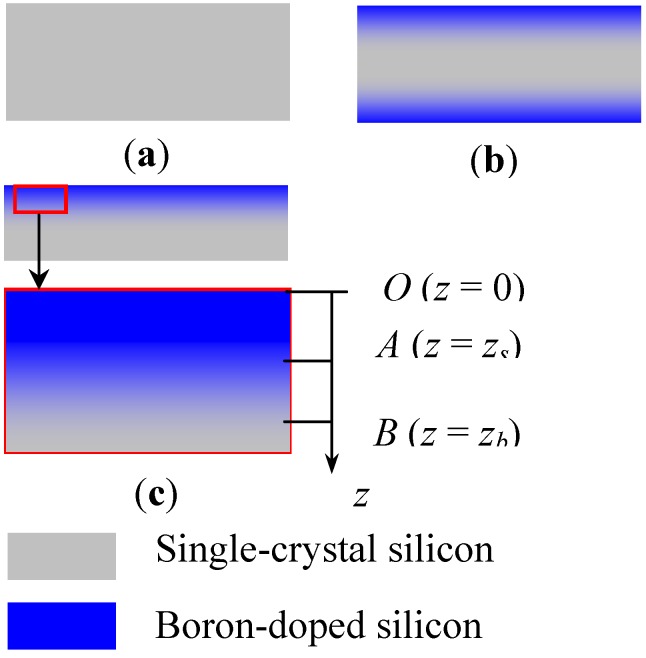
Process of boron diffusion in silicon. (**a**) Silicon wafer. (**b**) Double-sided boron doping. (**c**) Boron profile of thinned wafer.

Figure 1 shows the silicon self-stopped etching process. The substrate material for diffusion is a polished P-type (100) silicon wafer. Figure 1a shows the cross section of the wafer. The boron diffusion includes the predeposition process and the drive-in process. The former is carried out in a diffusion furnace in an N_2_ atmosphere at a temperature of 1050 °C. The latter is carried out on the diffused wafer after predeposition in an O_2_ atmosphere at a temperature of 1180 °C. Boron diffusion occurs on both sides of the wafer, as shown in Figure 1b, and the diffusion depth depends on both the predeposition and the drive-in processes. Only one side of the boron-doped wafer is used for structure fabrication in MEMS, thus a thinning process is first applied; Figure 1c shows the thinned wafer. The curvature behavior is produced after wafer thinning, which is generally realized by either milling or etching, according to the practical requirements. The level of thinning determines the curvature level, which physically depends on the boron distribution in the silicon wafer.

The boron doping concentration in silicon is assumed to be a Gaussian function of the depth according to the solid diffusion mechanism, which depends on factors such as the solid solubility, diffusion temperature, and diffusion time [14]. The total thickness of silicon after self-stopped etching is determined by the etchant type and boron concentration; therefore, the etching characteristics can be used to identify the profile of boron atoms in the silicon. As shown in Figure 1c, the coordinate’s axis denotes only the depth; the boron concentration at point *O* (surface) is unknown after the drive-in process, but that the depth can be determined by two separate, self-stopped, etching processes using different etchants [15]. One etchant is *ψ* (HF:HNO_3_:CH_3_COOH = 1:3:8), the stop condition of which is at a boron atom concentration of 1 × 10^17^ cm^−3^ (point *B*). The other etchant is 30%–40% KOH, the stop condition of which is at a boron atom concentration of 5 × 10^19^ cm^−3^ (point *A*). Therefore, the Gaussian functions (*f*(*z*)) of the boron profile with the boundary conditions for points *A* and *B* are given as *f* (*z_s_*) = 5 × 10^19^ cm^−3^ and *f* (*z_b_*) = 1 × 10^17^ cm^−3^.

For simplification, the relative concentration is defined as the ratio of boron atoms to silicon atoms per unit volume. The boron profile in silicon can be expressed as:

(1) $$C\left(z\right)=\frac{f\left(z\right)}{f_{Si}\left(z\right)}=\frac{f\left(z\right)}{5\times 10^{22}}$$

with the boundary conditions *C* (*z_s_*) = 1 × 10^−3^ and *C* (*z_b_*) = 2 × 10^−6^.

Compared with undoped silicon, heavily boron-doped silicon has a 0.8% higher thermal expansion coefficient [16] and a 20%–30% higher Young’s modulus [17]. Moreover, Pauling’s covalent radius of boron is approximately 25% smaller than that of silicon [5]; thus, the volume of the doped parts tends to shrink relative to that of the undoped parts, and the shrinking rate varies with the doping concentration. Therefore, boron-doped silicon can be modeled as a composite material solid structure having a Young’s modulus that follows a Gaussian function through the thickness. Buckling occurs when the structure is subjected to a nonuniform strain through the thickness. Generally, the nonuniform strain is caused by the residual stresses, crystalline defects, and volume shrinkage induced by doping gradients. The proposed buckling model only considers the shrinkage strain because the influence of the thermal stress and crystalline defects are neglected owing to the slight difference between the thermal expansion coefficients and the annealing process [5].

Consider a rectangular plate made of boron-doped silicon with the cross section shown in Figure 1c. Young’s modulus and volume shrinkage strain of the plate can be assumed as:

(2) $$\left\{ \begin{array}{l} E\left(z\right)=E_{Si}+C\left(z\right)\frac{E_{\max}-E_{Si}}{C_{\max}}\\ \varepsilon_{s}\left(z\right)=C\left(z\right)\frac{\varepsilon_{\max}}{C_{\max}} \end{array}\right.$$

where *C*_max_ (= 8 × 10^−3^) is the maximum relative concentration determined by the solid solubility of boron in silicon, *E*_max_ (= 210–220 GPa) and *ε*_max_ (= −2.5 × 10^−4^) are Young’s modulus and shrinkage strain to *C*_max_, respectively, and *E_Si_* (= 169 GPa) is Young’s modulus of silicon. The values of *E*(*z*) and *ε_s_*(*z*) can be calculated under the condition that the Young’s modulus and the shrinkage strain of boron-doped silicon are both proportional to the boron concentration.

**Figure 2:**
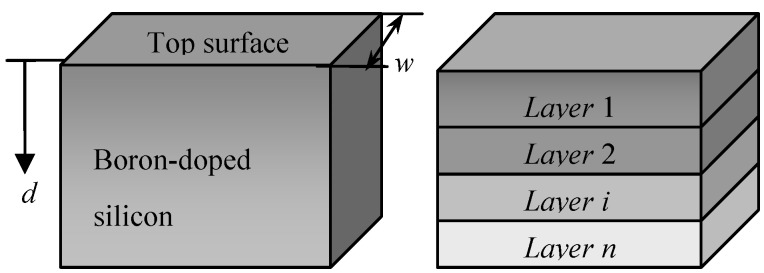
The solid structure is divided into a multilayer model.

**Figure 3:**
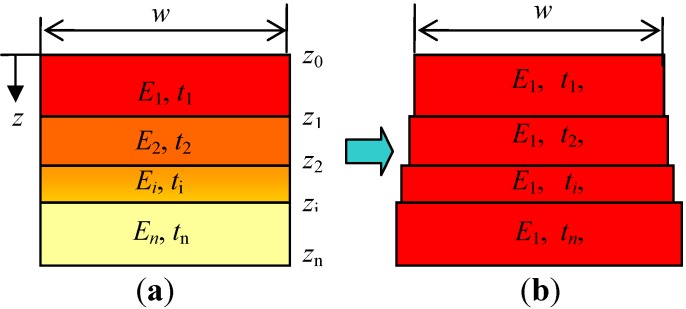
Multilayer model with nonuniform Young’s modulus is equivalently substituted by one with uniform modulus* E*_1_ with changed widths: (**a**) Multilayer model and (**b**) Equivalent model.

The nonuniformity induced by doping along the depth can be approximated by a multilayer model based on the finite element method because the infinite thin layer, which is vertical to the depth direction *z*, has uniform properties in the plane. The boron-doped silicon structure can therefore be modeled as a compound, formed by a group of thin layers glued together, as shown in Figure 2. The solid on the left is divided into an *n*-layer structure and shown on the right. Each layer has a uniform property that differs from that of other layers. Figure 3a shows the cross section of the multilayer model, where *w* is the width and *E*_i_ and *t*_i_ are Young’s modulus and thickness of layer *i*, respectively. The buckling of the structure produces a neutral plane that undergoes bending deformation but not extended deformation. The position of the plane can be determined by an equivalent transformation of the resistance to bending, as shown in Figure 3b. Except for the first layer, all layers are replaced with a material with the same Young’s modulus as the first layer. Under the condition of equivalent resistance to bending, it can be assumed that the thickness *t*_i_ of all the layers is not changed. Consequently, the width of layer *i* can be expressed as:

(3) $$w_{i}=\frac{E_{i}}{E_{1}}w$$

Therefore, the central principal axis of the cross section can be expressed as:

(4) $$\begin{array}{l} z_{c}=\frac{\sum S_{y}}{\sum A}=\frac{\frac{1}{2}\sum_{i=1}^{n}w_{i}t_{i}^{2}+\sum_{i=2}^{n}\left(w_{i}t_{i}\sum_{j=1}^{i-1}t_{j}\right)}{\sum_{i=1}^{n}w_{i}t_{i}}\\ \;=\frac{\frac{1}{2}\sum_{i=1}^{n}E_{i}t_{i}^{2}+\sum_{i=2}^{n}\left(E_{i}t_{i}\sum_{j=1}^{i-1}t_{j}\right)}{\sum_{i=1}^{n}E_{i}t_{i}} \end{array}$$

According to the derivation in earlier studies [18,19,20,21] and the material mechanics theory, the strain along the depth of the model can be expressed as:

(5) $$\varepsilon =\varepsilon_{0}+\frac{z_{c}-z}{r}$$

where *ε* is the total strain; *ε*_0_, the uniform strain; *z*, the coordinate along the depth; and *r*, the radius of curvature of the neutral plane. Therefore, the stress in the layers can be expressed as:

(6) $$\sigma =E_{i}\left(\varepsilon -\varepsilon_{i}\right)$$

where *E_i_* is Young’s modulus and *ε_i_*, the shrinkage strain induced by the doping of layer *i*.

According to the relationship in static mechanics, the resultant force and moment of each layer can be expressed as:

(7) $$\left\{ \begin{array}{l} F_{i}=\int_{z_{i-1}}^{z_{i}}\sigma wdz\\ M_{i}=\int_{z_{i-1}}^{z_{i}}\sigma w\left(d_{c}-z\right)dz \end{array}\right.$$

where *z_i_* is the coordinate point located at the interface of two adjacent layers, as shown in Figure 3a.

Under a free state, such as free-standing and with simple supports, the resultant force and moment in the total cross section is equal to zero:

(8) $$\left\{ \begin{array}{l} F=\sum_{i=1}^{n}F_{i}=\sum_{i=1}^{n}\int_{z_{i-1}}^{z_{i}}wE_{i}\left(\varepsilon -\varepsilon_{i}\right)\text{d}z=0\\ M=\sum_{i=1}^{n}M_{i}=\sum_{i=1}^{n}\int_{z_{i-1}}^{z_{i}}wE_{i}\left(\varepsilon -\varepsilon_{i}\right)\left(z_{c}-z\right)\text{d}z=0 \end{array}\right.$$

Substituting Equations (5) and (6) into (8) gives

(9) $$\left\{ \begin{array}{l} \sum_{i=1}^{n}\int_{z_{i-1}}^{z_{i}}E_{i}\left(\varepsilon_{0}+\frac{z_{c}-z}{r}-\varepsilon_{i}\right)\text{d}z=0\\ \sum_{i=1}^{n}\int_{z_{i-1}}^{z_{i}}E_{i}\left(\varepsilon_{0}+\frac{z_{c}-z}{r}-\varepsilon_{i}\right)\left(z_{c}-z\right)\text{d}z=0 \end{array}\right.$$

Assuming that Poisson’s ratio is constant, the radius of the curvature induced by the nonuniform shrinkage strain can be expressed as:

(10) $$r=\frac{\sum_{i=1}^{n}E_{i}\left[2\left(z_{i}^{3}-z_{i-1}^{3}\right)-3z_{c}\left(z_{i}^{2}-z_{i-1}^{2}\right)\right]}{3\sum_{i=1}^{n}E_{i}\left(\varepsilon_{0}-\varepsilon_{i}\right)\left(z_{i}^{2}-z_{i-1}^{2}\right)}$$

The radius of curvature is determined by Young’s modulus and shrinkage strain of each layer, and it is not affected by the dimensions of the structure when the boundary condition of the model satisfies *F* = 0 and *M* = 0.

## 3. Experimental Verification

The curvature of inertial sensors induced by boron doping was first observed by optical interferometry in [5]; however, this study did not report the cause of the curvature. Based on the above derivation, the radius of curvature is independent on the in-plane dimensions of the fabricated structures and is dependent on the boron-doping level and thickness. Therefore, in the experimental verification, we aim to investigate the effects of the dimensions and thickness on the sensor curvature.

**Figure 4:**
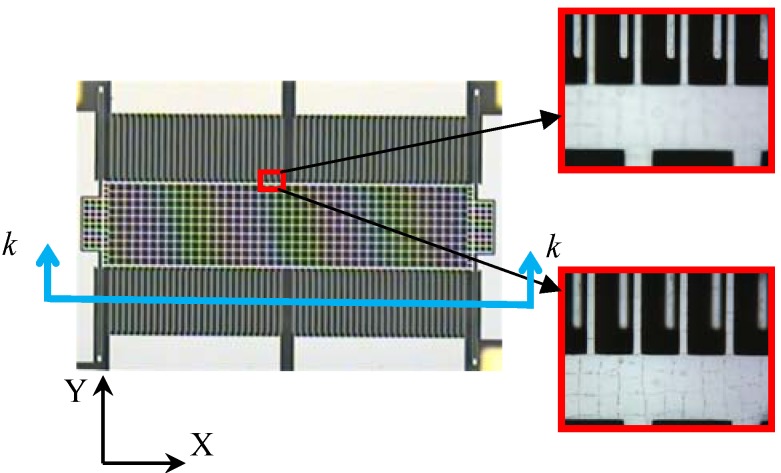
Observed buckling phenomenon under microscopy.

The experiment was performed using accelerometer sensors, fabricated through the bulk silicon- dissolved wafer process. The out-of-plane mismatch of fixed and movable fingers of the proof mass was observed through calibrated microscopy, as shown in Figure 4. Two groups of fingers were not in the same focal plane. The height differences among the mismatched fingers were investigated by surface profilometry. The differences were recognized as the buckling values of the proof mass block at the observed area because the fixed fingers were attached to the substrate and the movable fingers were attached to the proof mass, which was symmetrically supported by four U-type beams. The boundary condition of the mass plane can be set as a simple support that satisfies *F* = 0 and *M* = 0 when buckling occurs, and the buckling values along the X-axis in Figure 4 can be expressed as:

(11) $$z_{buckling}=\sqrt{r^{2}-{\left(X-\frac{L}{2}\right)}^{2}}-\sqrt{r^{2}-{\left(\frac{L}{2}\right)}^{2}}$$

where *z_buckling_* is the buckling value at coordinate X and *L*, the length of a mass block.

**Figure 5:**
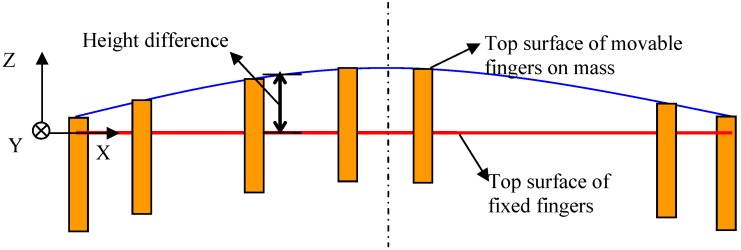
Section *k* – *k* of mass block shown in Figure 4.

**Figure 6:**
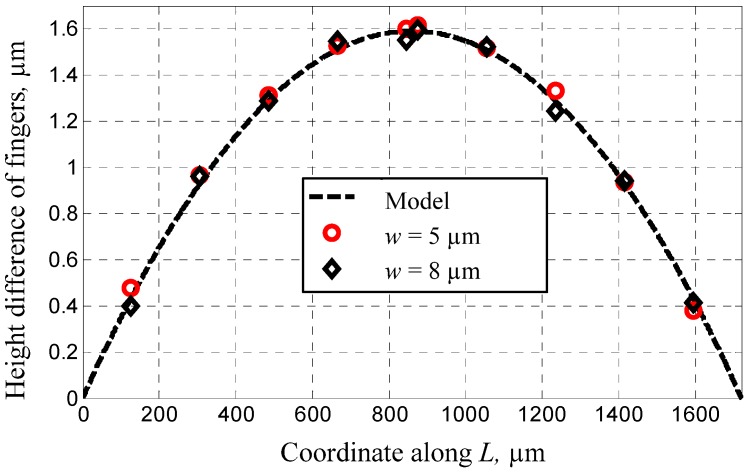
The height difference of comb fingers along the X-axis with different U-type beams.

**Figure 7:**
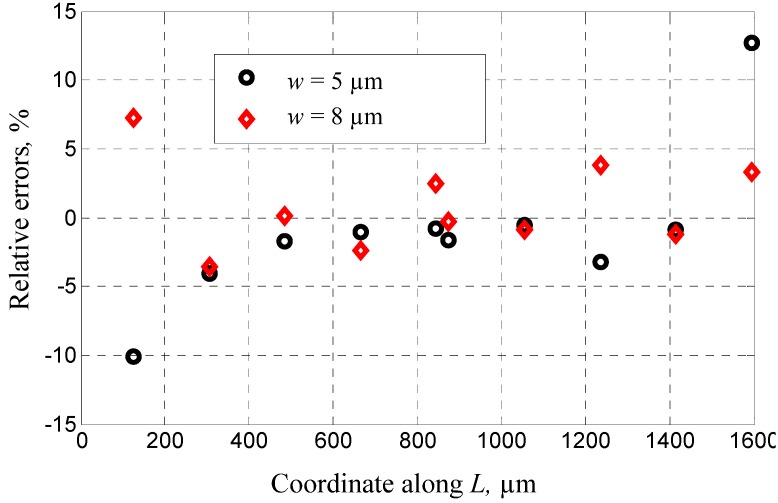
The relative errors of buckling values from experiments and models.

**Figure 8:**
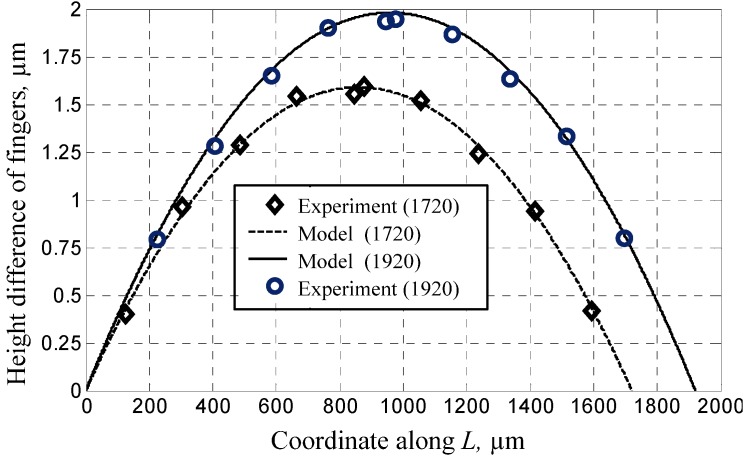
The buckling value with different lengths of mass block.

Five groups of sensors were fabricated by using the dissolved wafer process and measured through microscopy and surface profilometry. The main dimensions are listed in Table 1. *w* represents the width of U-type beams and *L*, the length of the mass block along the X-axis shown in Figure 4. The measurements of Groups 1 and 2 aim to derive the buckling curve and its dependence on the supporting beams. Therefore, the height differences among the comb fingers on the mass block along the X-axis are observed and their definitions are shown in Figure 5. The results shown in Figure 6 and the relative errors shown in Figure 7 indicate that the buckling value of the mass block is only slightly influenced by the width of the U-type beams because the resistance created by the beams against the deformation of the block is slightly related to the shrinkage strength induced by the covalent bond. Based on this conclusion, the dependence of the buckling value on the length of the mass block is studied by comparing Group 2 with Group 5. The results are shown in Figure 8. The curves of the measurement data from the two groups have the same radius of curvature. This coincides with the result obtained from the theoretical model, which is equal to 2.33 × 10^5^ μm. The coincidence of the results is produced by the uniform concentration of boron diffusion in the bulk silicon on the plane parallel to the silicon surface as well as the equal shrinkage strain in the structures with the same thickness.

**Table 1:** Geometrical parameters of sensors.

Group	Thickness		Sensor dimensions	
	*d_s_*, µm	*d_b_*, µm	*L*, µm	*w*, µm
1	42	83	1720	5
2	42	83	1720	8
3	42	97	1720	8
4	31	83	1720	8
5	42	83	1920	8

The final experiment focuses on the influence of diffusion conditions on the buckling values. As described previously, the diffusion process consists of predeposition and drive-in processes, both of which determine the thickness of boron-doped silicon and the sensor structure, and are influenced by diffusion conditions including the temperature, time, and gas environment. The sensors in Groups 2–4 have the same layout but are fabricated under different process conditions. The thicknesses *d_s_* and* d_b_* are measured by spreading the resistance instrument based on the self-stop etching mechanism described in Section 2. The largest height difference that occurs at the middle finger of each group is shown in Table 2. The results from both the model and the experiment indicate that the height difference, or buckling value of sensors with the same structure thickness, decreases with the increase in boron-doping depth because the Gaussian curve of the higher depth is flatter than that of the lower depth. Furthermore, the buckling of thin sensors under the same boron-doping depth has a smaller value because the curve of the Gaussian function near the peak value is flat. Consequently, a small concentration gradient is obtained.

**Table 2:** The largest buckling value with different diffusion depths.

Group	Thickness		Buckling value, µm	
	*d_s_*, µm	*d_b_*, µm	Experiment	Model
2	42	83	1.55	1.59
3	42	97	1.31	1.42
4	31	83	0.90	1.03

## 4. Conclusions

The curvature of the sensor in the accelerometer created through the BDWP is investigated. The multilayer model and corresponding analytical results are established to identify the buckling value of the proof mass block, which has a larger boron-doping thickness than that of the diaphragm or membrane studied in surface micromachining. Owing to the extended time required for the predeposition and drive-in processes of a thick boron-doped structure, the defects and thermal stress in the doped parts are less than those in the surface-machined structure. Therefore, the proposed model only aims to investigate the effects of volume shrinkage and Young’s modulus variation on the buckling of the bulk silicon structure. The results indicate that the model is effective in predicting the buckling value of the boron-doped structure, as verified through a series of experiments carried out by microscopy and using a spreading resistance instrument according to the diffusion mechanism of boron in silicon.

The buckling value of the sensor is determined not only by the thickness of boron-doped silicon in the predeposition process, but also by the conditions employed in the drive-in process. According to the results, only a smaller buckling value can be acquired through suitable predeposition and drive-in times. When the boron-doped depth is invariable, the buckling value of the thin sensor structure is smaller than that of the thick structure. When the sensor structures have equal values, a greater doping depth leads to a smaller buckling value. The buckling is determined by the boron concentration in silicon and is slightly influenced by the supporting beams, the stiffness of which is relatively low. Moreover, the radius of curvature of buckling is determined by the boron concentration in silicon and not by the geometrical parameters of the plane. Therefore, the buckling value can be ignored when the plane dimensions are relatively small.
